# Impact of Estrogen Withdrawal and Replacement in Female Mice along the Intestinal Tract. Comparison of E2 Replacement with the Effect of a Mixture of Low Dose Pollutants

**DOI:** 10.3390/ijerph18168685

**Published:** 2021-08-17

**Authors:** Claudie Pinteur, Benoit Julien, Nathalie Véga, Hubert Vidal, Danielle Naville, Brigitte Le Magueresse-Battistoni

**Affiliations:** Univ-Lyon, CarMeN Laboratory, INSERM U1060, INRAE U1397, Université Claude Bernard Lyon1, F-69310 Pierre-Bénite, France; claudie.pinteur@univ-lyon1.fr (C.P.); julien.benoit@outlook.fr (B.J.); nathalie.vega@univ-lyon1.fr (N.V.); hubert.vidal@univ-lyon1.fr (H.V.); navilledanielle@gmail.com (D.N.)

**Keywords:** ovariectomy, estradiol replacement, gut-liver tissue axis, high-fat, high-sucrose diet, mixture of pollutants, metabolic disorders

## Abstract

Postmenopausal women represent a vulnerable population towards endocrine disruptors due to hormonal deficit. We previously demonstrated that chronic exposure of ovariectomized C57Bl6/J mice fed a high-fat, high-sucrose diet to a low-dose mixture of chemicals with one dioxin, one polychlorobiphenyl, one phthalate, and bisphenol A triggered metabolic alterations in the liver but the intestine was not explored. Yet, the gastrointestinal tract is the main route by which pollutants enter the body. In the present study, we investigated the metabolic consequences of ovarian withdrawal and E2 replacement on the various gut segments along with investigating the impact of the mixture of pollutants. We showed that genes encoding estrogen receptors (Esr1, Gper1 not Esr2), xenobiotic processing genes (e.g., Cyp3a11, Cyp2b10), and genes related to gut homeostasis in the jejunum (e.g., Cd36, Got2, Mmp7) and to bile acid biosynthesis in the gut (e.g., Fgf15, Slc10a2) and liver (e.g., Abcb11, Slc10a1) were under estrogen regulation. Exposure to pollutants mimicked some of the effects of E2 replacement, particularly in the ileum (e.g., Esr1, Nr1c1) suggesting that the mixture had estrogen-mimetic activities. The present findings have important implications for the understanding of estrogen-dependent metabolic alterations with regards to situations of loss of estrogens as observed after menopause.

## 1. Introduction

Over the past decades, the prevalence of metabolic-related diseases such as obesity and type 2 diabetes has reached epidemic proportions worldwide. This represents major public health challenges of the 21st century. According to the World Health Organization [1], there are around 2 billion adults overweight, of those 650 million are considered to be affected by obesity (BMI ≥ 30 kg/m^2^). Obesity and metabolic diseases reflect an energy imbalance of multifactorial causes, including an environmental origin, according to convincing epidemiological data and experimental studies [2,3,4,5]. Endocrine disruptors are of particular concern since they can interfere with any hormonal action at low doses with non-monotonic dose-response curves [6,7]. Therefore, periods of high sensitivity to hormonal signals such as during development, puberty, and gestation, but also periods when sex steroid levels drop such as during aging represent periods of high vulnerability to endocrine disruptors.

Menopause is characterized by a constant decline in ovarian function and is accompanied by several pathologies such as metabolic syndrome (MetS) [8], of which abdominal obesity is the dominant MetS feature in women [9]. MetS is a major public health problem since it increases the risk of cardiovascular disease, stroke and type 2 diabetes that affect the overall quality of life causing social and psychological difficulties and imposing a major economic burden on societies [10]. In postmenopausal women, the prevalence of MetS increases varying between 30 and 70% compared to 14-45% in younger women, depending on ethnicity and other confounding factors such as lifestyle and eating habits [11]. Since women live at least one-third of their life after menopause, it is important to understand the mechanisms behind the development of metabolic diseases occurring during the menopausal transition in order to design prevention strategies.

Although imperfect, ovariectomy is a very common and accepted model developed in rodents to mimic post-menopausal conditions. Indeed, ovariectomized rodents develop the majority of MetS risk factors such as insulin resistance, visceral obesity, and inflammation [12], especially if the mice are fed with a high-fat diet since it accelerates the progression of menopausal symptoms compared to a low-fat diet [13]. Interestingly, previous studies suggested that ovariectomy can modulate intestinal permeability and inflammation and is accompanied by a state of dysbiosis associated with MetS [12], favoring the development and progression of non-alcoholic fatty liver disease (NAFLD) through the gut-liver axis [14]. However, the intestine has been little explored in mice exposed to pollutants [15,16] and not at all in ovariectomized mice. Yet, the gut is the second detoxifying tissue after the liver expressing various xenobiotic receptors and processing genes [17].

In the present study, we took benefit of an in vivo protocol previously carried out in female mice allowing the exploration of the metabolic consequences of ovarian withdrawal and E2 replacement along with investigating the impact of a mixture of pollutants exhibiting estrogen-mimetic properties [18,19]. The mixture consisted of archetypal endocrine disruptors among which only bisphenol A (BPA) is considered as an xenoestrogen [7]. They were each present at a dose close to the tolerable daily intake (TDI) dose as defined by the European Food Safety Agency (EFSA), and which is derived from the low or non-observed adverse effect levels (LOAEL/NOAEL) determined in experimental studies [20]. Specifically, it contained a dioxin and polychlorinated biphenyl (PCB153), two persistent chemicals that bioaccumulate in the food chain being particularly present in fatty foods of animal origin (meat, milk, fish) and characterized by half-lives of several years in humans [21]. The mixture also contained two short-lived chemicals, a phthalate and BPA massively used in plastic goods, the leaching from packaging causes contamination resulting in chronic exposure [22,23]. Interestingly, the mixture of pollutants triggers sex-dependent metabolic alterations in the liver and adipose tissue of chronically exposed mice, suggesting an interaction with estrogen signaling [24,25].

The overall objective of the present investigation was to identify changes in intestinal gene expression caused by exposure to low doses of pollutants that could act as early markers of chronic adverse effects, in order to better understand the mechanisms of toxicity and the role of estrogen. To this end, we undertook an extensive qPCR analysis of genes encoding estrogen receptors, xenobiotic processing genes, and genes related to nutrient uptake, gut permeability, and bile acid biosynthesis, using samples collected from different regions of the intestinal tract.

## 2. Materials and Methods

### 2.1. Experimental Design

C57BL/6 female mice of 4 weeks of age were purchased from Envigo laboratories (Gannat, France), housed in polypropylene cages at 21 °C with a normal light-dark cycle and free access to water (polypropylene bottles) and standard chow (LASvendi-16R; Soest, Germany). After a 1-week acclimatization period, mice were fed a high-fat, high-sucrose (HF) diet (from Envigo, MD.99249). One group of mice was fed a HF diet containing a mixture of pollutants, as described in [24]. Specifically, the mixture of pollutants was made of 2,3,7,8-tetrachlorodibenzo-p-dioxin (TCDD, CAS no. 1746-01-6; LGC-Promochem, Molsheim, France), polychlorinated biphenyl (PCB) 153 (CAS no. 35065-27-1), bisphenol A (BPA, CAS no. 80-05-7), and di-[2-ethylhexyl]-phthalate (DEHP, CAS no. 117-81-7) (all from Sigma-Aldrich, Saint-Quentin Fallavier, France). Pollutants were dissolved in dimethylsulfoxide (DMSO) and diluted in corn oil. Pollutant-free diet contained the same amount of DMSO (final concentration of 0.02% per kg of HF diet) and corn oil as the pollutant-containing food. The HF diets (with or without pollutants) contained 63% fat, 23.6% carbohydrate, and 13.4% protein in kcal, as detailed previously [19,24]. Each pollutant was used at the TDI dose (DEHP, BPA, TCDD) or four times the TDI dose (PCB153). Preparation of the contaminated food is presented in Table S1.

By 7 weeks of age, mice were either sham-operated (Sham) or underwent a bilateral ovariectomy (Ovx) followed by the implantation under the neck skin of Silastic implants filled either with vehicle or with 50 µg of 17β-estradiol-3-benzoate, E2 (Sigma-Aldrich, Saint-Quentin Fallavier, France) in 30 µL of sesame oil. The procedure has been described previously [19]. Implants were renewed at 13 weeks of age since the E2 release was only effective for 6 weeks based on body weight monitoring (data not shown). Uteri were also weighed at the end of the experiment (i.e., 20 weeks of age), to validate ovariectomy and E2 supplementation [19]. Together, four groups of 6 to 8 mice per group were generated. It included sham mice fed an HF diet with no added pollutants (Sham); Ovx mice fed an HF diet with no added pollutants (Ovx); Ovx mice treated with E2 fed an HF diet with no added pollutants (Ovx+E2); and Ovx mice fed an HF diet containing the mixture of pollutants (Ovx+poll) (Table S1). All procedures were performed with the approval of the Regional Committee of Ethics for Animal Experiments (CECCAP) registered under number C2EA15 by the French Ministry of Higher Education and Research in accordance with European directive 2010/63. The experimental protocol received the number CECCAP_LS_2014_004.

### 2.2. Blood and Tissue Collection

At 20 weeks of age, 6-h fasted mice were euthanized by cervical dislocation. Liver, adipose tissues, and gut segments were quickly dissected and snap-frozen in nitrogen liquid. Once dissected, gut segments (i.e., the jejunum, ileum, and colon) were rinsed with saline and frozen in liquid nitrogen.

### 2.3. RNA Extraction and Gene Expression Analyses

Total RNA was extracted from frozen tissues using the TRI Reagent (Applied Biosystems, Courtaboeuf, France). RNA integrity was determined using the Agilent 2100 Bioanalyzer and RNA 6000 Nano Kit (Agilent Technology, Massy, France). After reverse transcription using the prime Script RT Reagent kit (Takara Bio Europe SAS, Saint-Germain-en-Laye, France), cDNAs were analyzed by real time PCR using the SYBR Premix Ex Taq TM (Takara) in the presence of specific primers specifically designed to encompass an intron (Table S2), as described previously [26]. Data were normalized using housekeeper genes which differ according to the tissues. They encode TATA-Box binding protein (Tbp) for the gut segments and adipose tissues, and beta-glucuronidase (Gusb) for the liver.

### 2.4. Statistical Analyses

All statistical analyses were performed using one-way ANOVA, followed by the Bonferroni multiple comparison post-hoc test. Results were expressed as mean ± SEM and differences were considered significant at *p* < 0.05 using the group (Ovx) as reference.

## 3. Results

### 3.1. Impact of Estrogens and of the Pollutant Mixture on the Expression of the Estrogen Receptors in Various Intestinal Segments

**Figure 1:**
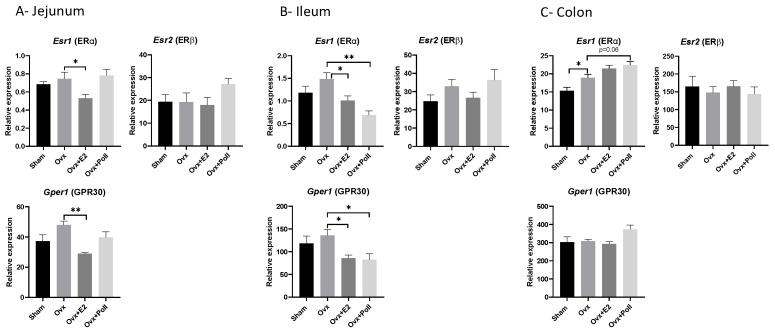
Effect of ovariectomy alone (Ovx) or with 17β-estradiol (E2) supplementation (Ovx+E2) or with exposure to pollutants (Ovx + Poll) on the expression of genes encoding the estrogen receptors ERα, ERβ, GPR30 in various segments of the gut: The jejunum (**A**), the ileum (**B**), and the colon (**C**). Values are means ± SEM with *n* = 6–8. ** *p* < 0.01; * *p* < 0.05. Sham: Sham-operated mice.

To better picture the consequences of ovariectomy on the gut, we first quantified the expression levels of the estrogen receptors in the jejunum, ileum, and colon as previously performed in the liver and adipose tissues of the same groups of female mice [19]. We observed that the expression levels of Esr1 and Esr2 genes encoding the nuclear estrogen receptor (ER) α and β, respectively, and Gper1 encoding the G-protein–coupled estrogen receptor 1 (GPR30) differ depending on the intestinal regions, with the highest level of expression of all three receptors in the colon, then the ileum and jejunum (Figure 1). Ovariectomy significantly affected Esr1 mRNA levels only in the colon (positively), while E2 replacement resulted in reduced mRNA levels in the jejunum and ileum of both Esr1 and Gper1 as compared to Ovx mice. Esr2 mRNA levels did not change between groups, regardless of the gut segment (Figure 1). Exposure to pollutants had an inhibitory effect on mRNA levels of Esr1 and Gper1 mRNA levels in the ileum (Figure 1). An upward trend in Esr1 mRNA levels (*p* = 0.06) was observed in the colon of Ovx mice exposed to pollutants compared to Ovx mice (Figure 1). Thus, exposure to pollutants triggered effects similar to those resulting from E2 replacement, suggesting that the mixture might have estrogen-mimetic activities in the jejunum and ileum.

### 3.2. Impact of Estrogen Withdrawal/Replacement and Pollutant Mixture on the Expression of Xenobiotic Processing Genes

**Figure 2:**
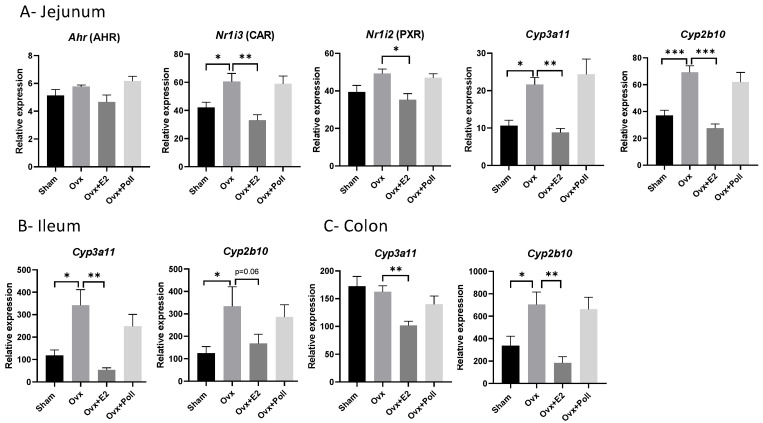
Effect of ovariectomy alone (Ovx) or with 17β-estradiol (E2) supplementation (Ovx+E2) or with exposure to pollutants (Ovx + Poll) on the expression of genes encoding xenobiotic processing genes including the xenobiotic receptors (AHR, PXR, and CAR) and the CYP3A11 and CYP2B10 in the jejunum (**A**); the CYP3A11 and CYP2B10 in the ileum (**B**) and the colon (**C**). Values are means ± SEM with *n* = 6–8. *** *p* < 0.001; ** *p* < 0.01; * *p* < 0.05. Sham: Sham-operated mice.

Preceding reports indicated that the pollutants of the used mixture can interact with xenobiotic receptors [27,28]. We focused on the expression levels of genes encoding the aryl hydrocarbon receptor (Ahr), the constitutive androstane receptor CAR (Nr1i3), and the pregnane xenobiotic receptor PXR (Nr1i2). We observed that Ahr mRNA levels did not vary between groups regardless of the intestinal segment (Figure 2 and Figure S1). Regarding the expression of the Nr1i3 gene, the jejunum responded to estrogen withdrawal with a significant increase not detected in ovariectomized mice given to E2 implants. Ovariectomy did not significantly affect Nr1i2 mRNA levels, which were significantly decreased in E2-replaced Ovx mice compared to Ovx mice (Figure 2). No inter-group variation was detected in the ileum and colon, with respect to Nr1i2 and Nr1i3 mRNA levels (Figure S1). CAR and PXR immediate target genes, which encode the cytochrome P450 enzymes CYP2B10 and CYP3A11, respectively [29], were also surveyed. With the exception of Cyp3a11 mRNA levels in the colon, which were in the same range in Sham and Ovx mice, we observed upregulation of both Cyps in response to estrogen withdrawal which was avoided in E2-replaced Ovx mice in the jejunum, ileum, and colon (a trend in the ileum) (Figure 2). As observed with xenobiotic receptors, exposure to pollutants did not cause any change in the expression levels of Cyps (Figure 2).

To complete the study, we compared these results in the gut with data from the liver. Contrasting the jejunum, we found that estrogen withdrawal caused downregulation of Ahr mRNA levels in the liver, an effect not evidenced in E2-replaced Ovx mice compared to Ovx mice. However, Nr1i2 and Nr1i3 mRNA levels did not fluctuate in the liver between groups. In addition, the expression of both hepatic Cyps was downregulated in Ovx mice (a trend for Cyp2b10) with further reduction in E2-replaced Ovx mice as compared to Ovx mice (Figure S2). Adipose tissues, which also express AHR but not PXR and CAR (Ref. in [5]) were investigated and Ahr mRNA levels fluctuated negatively with estrogens in the SAT as observed in the liver, while no significant effect was described in the VAT samples (Figure S2). It indicated that xenobiotic receptors were transcriptionally regulated in a distinct way depending on metabolic tissues.

### 3.3. Impact of Estrogens and of the Pollutant Mixture on the Expression of Genes Related to Gut Homeostasis in the Jejunum

**Figure 3:**
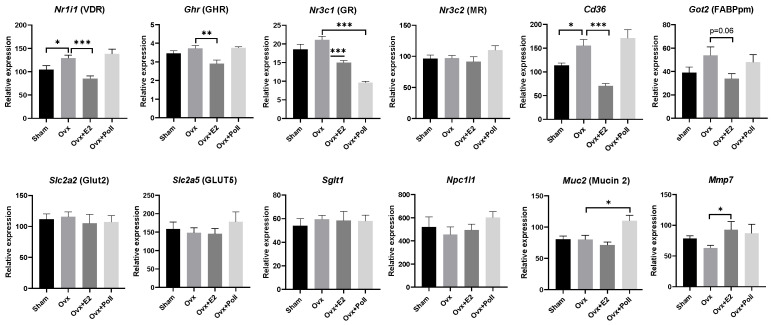
Effect of ovariectomy alone (Ovx) or with 17β-estradiol (E2) supplementation (Ovx+E2) or with exposure to pollutants (Ovx + Poll) on the expression of genes encoding various nuclear receptors, lipid and glucose transporters, and mucin 2 and MMP7 in the jejunum. Values are means ± SEM with *n* = 6–8. *** *p* < 0.001; ** *p* < 0.01; * *p* < 0.05. Sham: Sham-operated mice.

Then, we investigated the expression of various nuclear receptors which have been shown to cross-interact with estrogen signaling machinery including Nr1i1 encoding the vitamin D receptor [30], the growth hormone receptor (GHR) [31], and Nr3c1 and Nr3c2, encoding the gluco- and mineralo-corticoid receptors, respectively [32]. With the exception of Nr3c2, all receptors had their mRNA expression levels significantly decreased in E2-replaced Ovx mice compared to Ovx mice, suggesting that estrogens exerted a negative regulation. Consistently, Ovx resulted in increased mRNA levels of Nr1i1. Exposure to pollutants only affected Nr3c1 mRNA levels by decreasing mRNA levels as observed with E2 replacement (Figure 3).

It has been suggested that estrogens can alter the expression of genes encoding inflammatory markers, as well as those related to intestinal permeability thus impacting gut homeostasis [33]. To determine the regulatory roles of estrogens in the different intestinal segments, genes encoding different important metabolic genes were also analyzed by RT-qPCR. The jejunum is a major site for nutrient uptake but only genes involved in fatty acid uptake, as evidenced by measurements of mRNA levels of Cd36 and Got2 (encoding the fatty acid binding protein mFABP), was subject to estrogen regulation. Indeed, ovariectomy resulted in an increase in Cd36 levels, which was completely avoided in Ovx mice that received E2 supplementation. The data for Got2 were less significant and only a decreasing trend (*p* = 0.06) was observed between the Ovx and Ovx+E2 replacement groups (Figure 3). In contrast, the expression of the glucose transporter (i.e., the solute carrier genes Slc2a2 and Slc5a1 encoding Glucose Transporter 2, GLUT2, and sodium-dependent glucose cotransporter SGLT1, respectively), the fructose transporter (Slc2a5 encoding GLUT5), and the cholesterol transporter (Npc1l1 gene encoding the Niemann Pick C1 Like-1, NPC1L1) genes was not altered (Figure 3). Mucin 2, which is an indicator of goblet cells and the metalloproteinase MMP7 that converts inactive pro-defensins synthesized by Paneth cells to active molecules mediating host defense and homeostasis at the intestinal mucosal surface [34] were explored, as well. When compared to Ovx mice, we found that Mmp7 mRNA levels were significantly increased in Ovx mice E2-replaced and Muc2 mRNA levels were enhanced in Ovx mice exposed to the mixture of pollutants (Figure 3).

### 3.4. Impact of Estrogens and of the Pollutant Mixture on the Expression of Genes Related to Gut Homeostasis in the Ileum and Colon

**Figure 4:**
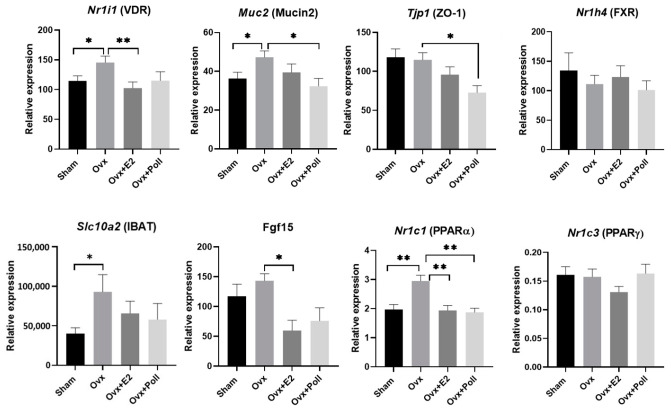
Effect of ovariectomy alone (Ovx) or with 17β-estradiol (E2) supplementation (Ovx+E2) or with exposure to pollutants (Ovx + Poll) on the expression of genes encoding various nuclear receptors, mucin2, IBAT, and FGF15 in the ileum. Values are means ± SEM with *n* = 6–8; ** *p* < 0.01; * *p* < 0.05. Sham: Sham-operated mice.

The various nuclear receptors quantified in the jejunum were also analyzed in the ileum and colon. None of them had the expression changed with estrogen deprivation with the exception of Nr1i1, in which mRNA levels fluctuated negatively with estrogen levels (Figure 4), as observed in the jejunum (Figure 3). As for jejunum, Mmp7 and Muc2 mRNA levels were also quantified. Other genes included markers of permeability involved in the intestinal barrier, specifically Tjp1, Cldn2, and Ocdn which encode zonula occludens 1 (ZO-1), claudin 2, and occludin, respectively. Additionally, genes encoding transcription factors involved in the differentiation of the different cell lineages in the intestine. For example, the protein atonal homog 1 (ATOH1) is essential for the formation of intestinal secretory cells (goblet, enteroendocrine, and Paneth cells). Neurogenin 3 (NGN3) is required for enteroendocrine differentiation. On this panel of genes, no significant impact on any of the measured genes was observed following ovariectomy and E2 replacement with the exception of Muc2 in the ileum and Tjp1 and Atoh-1 in the colon (Figure 4, Figures S3 and S4). Exposure of ovariectomized mice to the pollutant mixture decreased the levels of Muc2 and Tjp1 mRNA in the ileum compared to Ovx mice.

### 3.5. Impact of Estrogens and of the Pollutant Mixture on the Expression of Genes Related to Bile Acid Biosynthesis in the Gut and Liver

The synthesis of bile acids (BA) from cholesterol in the liver is strictly regulated by a negative feedback mechanism through a process known as enterohepatic circulation which refers to the circulation of BA from the liver to the bile, followed by entry into the small intestine, absorption by the enterocyte, and return to the liver through the portal vein. The complex scenario largely depends on the expression levels of specific bile acid efflux and uptake transporters within the liver and ileum, which are regulated by the farnesoid X receptor, FXR [35,36]. Therefore, we focused on a panel of genes dedicated to the process of BA biosynthesis, in the liver and ileum of the four groups of mice.

In the ileum, the expression of Nr1h4 encoding FXR did not vary between groups unlike Slc10a2 encoding the ileal sodium/bile acid cotransporter, also known as apical sodium–bile acid transporter (ASBT) and ileal bile acid transporter (IBAT). Specifically, the data showed significant upregulation in Ovx mice as compared to Sham mice, which was not demonstrated in the ileum of Ovx mice, whether E2-replaced or exposed to pollutants (Figure 4). In addition, fgf15 encoding fibroblast growth factor 15, which plays an important role in feedback inhibition of hepatic bile acid synthesis, has lowered mRNA levels in E2-replaced Ovx mice compared to Ovx mice. Ovx mice exposed to pollutants had levels in the same range as E2-replaced Ovx mice (Figure 4).

**Figure 5:**
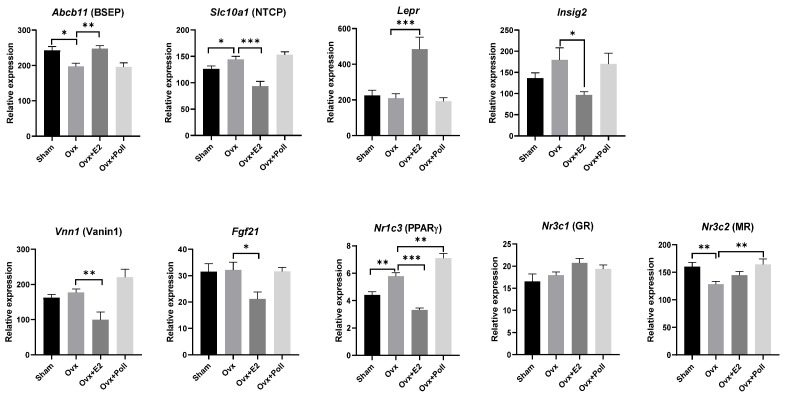
Effect of ovariectomy alone (Ovx) or with 17β-estradiol (E2) supplementation (Ovx+E2) or with exposure to pollutants (Ovx + Poll) on the expression of genes encoding various nuclear receptors and target genes, and BA transporters in the liver. Values are means ± SEM with *n* = 6–8. *** *p* < 0.001; ** *p* < 0.01; * *p* < 0.05. Sham: Sham-operated mice.

In the liver, ovariectomy caused a significant decrease in the mRNA levels of Abcb11 (Figure 5), which encodes the bile salt export pump (BSEP), an ATP-dependent transporter localized to the canalicular membrane and which directs the efflux of BAs from hepatocytes into the bile. Along with the decrease in Abcb11, we showed a significant increase in the levels of Slc10a1 mRNA (Figure 5), which encodes the sodium/bile acid co-transporter also known as the Na + -taurocholate co-transporter polypeptide (NTCP) or liver bile acid transporter (LBAT) and permits re-entry of bile salts in hepatocytes. E2 replacement in Ovx mice prevented BA accumulation in hepatocytes through enhancing Abcb11 and reducing Slc10a1 mRNA levels as compared to Ovx mice (Figure 5). Pollutants did not modify the expression pattern of the BA transporters, Abcb11, and Slc10a1 (Figure 5).

Moreover, we measured the expression levels of different genes related to cholesterol biosynthesis such as Lepr and Insig2, encoding the leptin receptor and the insulin-induced gene 2 [37,38]. While data showed no changes following ovariectomy, Lepr was strongly enhanced and Insig2 was strongly decreased in E2-replaced Ovx mice compared to Ovx mice. However, pollutant exposure did not affect these parameters (Figure 5).

Peroxisome proliferator-activated receptors (PPARs) especially PPARα also contribute to regulating BA synthesis, transport, and signaling [39]. In the ileum, Nr1c1 (PPARα) mRNA levels increased in Ovx mice compared to Sham mice unlike Nr1c3 (PPARγ) mRNA levels which did not change between groups. Interestingly, E2 replacement and pollutant exposure prevented the increase in Nr1c1 mRNA levels in Ovx mice (Figure 4). In the liver, Fgf21 and Vnn1 mRNA levels were decreased in line with the downregulation of Nr1c1 mRNA levels in the liver of E2-replaced Ovx mice [19]. However, pollutant exposure elicited no effects on these PPARα target genes in the liver (Figure 5). Furthermore, we investigated Nr1c3 in the liver, whose mRNA levels increased in Ovx mice compared to sham mice. E2-replaced Ovx mice showed levels equivalent to Sham mice unlike Ovx mice exposed to pollutants whose Nr1c3 mRNA levels were significantly higher than in Ovx mice (Figure 5). In the ileum, no variation in Nr1c3 mRNA levels was demonstrated between groups (Figure 4).

Finally, we explored in the liver, the expression levels of Nr3c1 and Nr3c2 encoding the gluco- and mineralo-corticoid receptors, respectively. Only Nr3c2 mRNA levels in the liver fluctuated between groups with downregulation in Ovx mice compared to Sham mice and significant upregulation in Ovx mice exposed to pollutants (Figure 5). Table 1 summarizes all the results on gene regulation by ovariectomy and/or E2 replacement and/or pollutant exposure.

## 4. Discussion

In the present study, we used a previously validated in vivo mouse protocol [19] to explore the metabolic consequences of ovarian withdrawal and E2 replacement on peripheral metabolic tissues, as well as to study the impact of a low-dose mixture of pollutants containing a dioxin, PCB153, DEHP, and BPA. The main objectives of this study were to complement the recently published data on liver and adipose tissues [19] by focusing on the different segments of the intestine: Jejunum, ileum, and colon. Indeed, the gut is the main route by which pollutants enter the body and it is the second detoxifying tissue after the liver [17]. In addition, estrogens have been shown to play important roles in the organization and architectural maintenance of the gut [40], to protect from intestinal inflammation [41] and contribute to bile acid biosynthesis through the enterohepatic circulation [14].

First, we studied the intestinal expression of a large range of receptors, not only estrogen receptors, but also xenobiotic receptors and receptors previously identified as interfering with the signaling machinery of estrogen receptors such as PPARs, VDR, GHR, and GR [32,42,43,44,45]. Indeed, the pollutants in the mixture were individually identified as metabolic disruptors [27,46,47] and estrogen-mimicking activities were demonstrated in the mixture [18,24,25]. Individually, BPA was originally described as pro-estrogenic due to its binding to estrogen receptors. However, modes of action involving a large repertoire of nuclear receptors including the PPARs and GR, as well as non-genomics modes of action closely related to metabolic health and body weight have also been described [2,48,49]. Phthalates, which are other short-lived chemicals used to soften plastics and to which humans are also chronically exposed, can bind to xenobiotic receptors and PPARs as described for BPA, and exert anti-androgenic effects [7,27]. Dioxins and PCBs, which belong to the group of persistent organic pollutants (POPs), can bind to AHR or interact with its signaling mechanisms such as PCB153, which do not bind to AHR. They may activate PXR and CAR as well, and interact with estrogen and thyroid signaling pathways, respectively [27,47,50,51,52,53].

We demonstrated differential estrogen receptor expression along the gastrointestinal tract with the highest level of expression of all three receptors in the colon, which was also the segment that reacted least to estrogen withdrawal or E2 supplementation. Among the three receptors, we observed that Esr2 mRNA levels did not fluctuate with estrogenic challenges regardless of the segment of the gut. Unlike Esr2, Esr1 and Gper1 responded similarly to E2 supplementation in the jejunum and ileum, at the mRNA level. However, these tissues are distinguished by their response to pollutant exposure since in the ileum but not in the jejunum, the mRNA levels of Esr1 and Gper1 are affected in response to exposure to pollutants. It is not currently clear why a mixture of pollutants would mimic the effects of E2 supplementation in the ileum but not in the jejunum. Importantly, exposure to pollutants did not affect the gene expression levels in the intestine of the xenobiotic receptors AHR, PXR, CAR, and of CYP3A11 and CYP2B10, prototypical targets of PXR and CAR, respectively. We previously observed that these genes were also unaffected by exposure to pollutants in the livers of the same mice [19]. Nonetheless, we herein showed that the mRNA expression of Nr3c1 (encoding GR) in the jejunum, Nr1c1 (encoding PPARα) in the ileum in addition to Esr1 and Gper1, and Nr1c3 (encoding PPARγ) and Nr3c2 (encoding MR) in the liver was affected by exposure to pollutants. These data confirm and extend previous findings showing that pollutants of the mixture can target PPAR and GR not only in the liver [27,53,54] but also in the gut in addition to estrogen receptors. Furthermore, with the exception of Nr1c3 in the liver, pollutant effects mimicked those induced by E2 supplementation in line with our previous findings that the mixture of pollutants had estrogen-mimetic activities [18,24,25]. Whether the Nr1c3 mRNA upregulation detected in the liver of ovariectomized mice exposed to the mixture of pollutants is correlated with the upward trend in liver TG described previously [19], will await further studies.

Unlike xenobiotic receptors, CYP3A11 and CYP2B10 mRNA expression was tightly regulated by estrogens in the various intestinal segments analyzed. As PXR and CAR were only regulated by estrogens in the jejunum and not in the ileum, colon, and even in the liver, it is likely that other nuclear receptors and/or cross-talk with other transcription factors contribute to the regulation of the expression of Cyp3a11 and Cyp2b10 in the ileum and colon. This may also indicate that PXR and CAR have functions other than the sensing of xenobiotics in the jejunum and that those functions which might be related to glucose and lipid metabolism as described in the liver [35,55,56] would be regulated by estrogens in the jejunum (at least in females). CAR and PXR, which cross-talk by sharing response elements and showing overlapping affinities for certain ligands, also share target genes such as CYP3A11 and CYP2B10 enzymes which show overlapping functions [29]. CYPs metabolize various steroids, fatty acids in addition to xenobiotics, and are subject to a complex regulatory scheme. For example, it has been reported that isoforms of CYP3A, which play an important role in the oxidation of endogenous steroids and toxic hydrophobic bile acids are modulated by exogenous and endogenous ligands not only via PXR and CAR but also via other receptors. These may be ERα [57], GR, FXR, VDR [58], and PPARα [59], some of them have been shown in the current study expression profiles as compatible with estrogen regulation in the jejunum and ileum. However, it is surprising that both estrogen withdrawal and E2 supplementation caused downregulation of CYP3A11 and CYP2B10 in the liver. It highlights that the regulation of these CYPs differ between the liver and the jejunum. The understanding of the estrogenic regulation of these genes which are also expressed in a sex-dimorphic manner [24,60] and regulated as well by glucocorticoids and GH [61] in the liver, warrants further studies. Such hormonal complexity probably explained why their regulation following ovariectomy and E2 replacement differed between the liver and gut (as observed in the present study) since these organs are distinctly regulated by hormones, not to mention estrogens [41,62,63].

Maintenance of the gut barrier function is essential for metabolic and immune health. It requires tightly regulated processes to cope with the transport, digestion, and absorption of nutrients that take place throughout the intestine from the duodenum to the colon and ensure intestinal immunity, food tolerance, and limited inflammation [64,65]. While ovariectomy induced obesity and triggered dysbiosis [66], we observed little changes in the mRNA expression levels of genes involved in epithelial cell differentiation and permeability throughout the gut segments with the exception of fatty acid transporters (Cd36 and Got2), Mmp7 in the jejunum, Muc2 in the ileum, and Tjp1 and Atoh1 in the colon. It has been demonstrated that transcription factors controlling intestinal differentiation [34] and intestinal permeability were deregulated in mice fed a high fat diet [65]. Worthy of note, temporal and regional changes in the in vivo intestinal permeability have recently been reported following loss of estrogen [33]. These authors demonstrated that while ovariectomy resulted in disturbed permeability accompanied with changes in tight-junction gene expression early after surgery, a normalization of the effects occurred over a period of 4 to 8 weeks that the authors suggested to be due to complex compensatory mechanisms. Since in the present study, the analysis was performed 13 weeks after surgery, compensatory mechanisms may also explain the absence of changes in the expression levels of genes involved in gut permeability. Determination of protein levels and immunohistochemical analysis at tight junctions would clarify whether or not there are changes in intestinal permeability under the different conditions studied.

Based on the findings that the Ovx mice given E2 supplementation exhibited an alteration in cholesterol metabolism possibly related to the over-expression of Esr1, Cyp7a1, and Hmgcr and the reduced expression of Nr1h4 (FXR) in the liver [19], we explored bile acid biosynthesis in both the liver and the gut. Indeed, overexpression of ERα in the liver triggers a loss in the fine tuning of negative feedback regulation of cholesterol synthesis [67] and HMG-CoA reductase and CYP7A1 are the rate-limiting enzymes of cholesterol metabolism and degradation in bile acids, respectively. On the panel of genes studied, transporters of bile acids were the most highly regulated both in liver and in ileum with profiles consistent with estrogens increasing efflux through BSEP from the liver but decreasing BA uptake in both the ileum (IBAT) and the liver (NTCP). In addition, E2 replacement caused a decrease of Fgf15 mRNA expression but no change in Nr1h4 (FXR) in the ileum, all highlighting deregulation of the BA biosynthesis process. Importantly, the mixture of pollutants produced effects similar to the E2 replacement on Slc10a2 and Fgf15 (although not significant), and on Nr1i1 (a trend) and Nr1c1 in the ileum. Since VDR and PPARα contribute to BA biosynthesis [39,68], further studies will have to be undertaken to determine whether pollutants act on VDR and/or PPARα to mediate their effects.

The relevance of our results must be compared with the dosage of the mixture in the range of the TDIs, thus not expected individually to generate a metabolic effect [16]. Consequently, the protocol set up here by demonstrating that several genes were affected (at least at the mRNA level) by the mixture of pollutants extended the concept of the cocktail effect. This concept states that the effect resulting from a mixture cannot be derived from the expected effect of a chemical analyzed individually, as reviewed [46,69]. The present findings may have important implications for the understanding of the estrogen-dependent metabolic alterations about situations of loss of estrogens as observed with menopause, especially if considering the worldwide context of exposure to chemicals.

## 5. Conclusions

The analysis of ovariectomized female mice supplemented with E2 or exposed to a mixture of pollutants with reported estrogen-mimetic activities [15,24,25] has broadened the knowledge on the estrogenic regulation in the intestine. We showed that genes encoding estrogen receptors (Esr1, Gper1 not Esr2), xenobiotic processing genes (e.g., Cyp3a11, Cyp2b10), and genes related to gut homeostasis in the jejunum (e.g., Cd36, Got2, Mmp7) and to bile acid biosynthesis in the gut (e.g., Fgf15, Slc10a2) and liver (e.g., Abcb11, Slc10a1) were under estrogen regulation. Moreover, we have provided new evidence on the metabolic consequences of exposure to pollutants, in particular in the ileum, which is strongly involved in the regulation of BA biosynthesis by FGF15 [70].

## Figures and Tables

**Table 1:** Summary of the findings. List of genes in which the mRNA gene expression level is altered by ovariectomy and/or E2 replacement or following pollutant exposure. “E2-like effects” mean that the effects exerted by the mixture of pollutants mimicked the effects shown in the group “Ovx + E2 replacement”. In this case, the name of the genes is in bold type.

	Genes Affected by Ovariectomy and/or E2 Replacement	Genes Affected by Pollutant Exposure Showing E2-like Effects
Jejunum	*Cd36; Cyp2b10; Cyp3a11; Esr1; Ghr; Got2; Gper1; Mmp7; Nr1i1; Nr1i2; Nr1i3; Nr3c1*	***Nr3c1;*** *Muc2*
Ileum	*Cyp2b10; Cyp3a11; Esr1; Fgf15; Gper1; Nr1c1; Nr1i1; Muc2; Slc10a2*	***Esr1; Gper1; Nr1c1; Muc2;*** *Tjp1*
Colon	*Atoh1; Cyp2b10; Cyp3a11; Esr1; Tjp1*	***Esr1***
Liver	*Abcb11; Ahr; Cyp2b10; Cyp3a11; Insig2; LepR; Nr1c3; Nr3c2; Slc10a1; Vnn1*	*Nr1c3; **Nr3c2***

## Data Availability

The data presented in this study are available upon request from the corresponding author.

## Supplementary Materials

The following are available online at https://www.mdpi.com/article/10.3390/ijerph18168685/s1. Table S1: Reference doses of the pollutants of the mixture and the dosage used; Table S2: List of the primers used for the qPCR analysis; Figures S1–S4.
