# Supplementary material for: Impact of Estrogen Withdrawal and Replacement in Female Mice along the Intestinal Tract. Comparison of E2 Replacement with the Effect of a Mixture of Low Dose Pollutants
Source: Int J Environ Res Public Health. 2021 Aug 17;18(16):8685. doi: 10.3390/ijerph18168685 (PMC8394409; doi:10.3390/ijerph18168685)

**Supplemental Table S1:** Reference doses of the pollutants of the mixture and the dosage used (all expressed per kg body weight and per day)

| Pollutants in the mixture | PCB153 | BPA | TCDD | DEHP |
|---|---|---|---|---|
| **NOAEL** | 40 µg | 5 mg | 2 ng | 5 mg |
| **TDI** | 20 ng | 4 µg | 1-4 pg | 50 µg |
| **Doses used** | 80 ng | 5 µg | 2 pg | 50 µg |

**Supplemental Table S2:** List of the primers used for the qPCR analysis.

| | | sequences: 5'->3' | |
|---|---|---|---|
| **gene** | **reference** | **sense** | **antisense** |
| | | | |
| ***Abcb11*** | NM_021022 | GCAGTCACACCCATCTACTC | TGGGAAGCATCTGTAGCAAG |
| ***Ahr*** | NM_013464.4 | TCA-TCT-GGT-TTC-CTG-GCA-ATG-AAT | ATA-AGC-TGC-CCT-TTG-GCA-TC |
| ***Atoh1*** | NM_007500 | CAG-CGA-TGA-TGG-CAC-AGA-AG | TGG-TTG-TCT-CAG-TTT-TCA-GG |
| ***Cd36*** | NM_001159558.1 | AAG-ATC-CAA-AAC-TGT-CTG-TA | GTC-CTG-GCT-GTG-TTT-GGA-GG |
| ***Cldn2*** | NM_016675 | CCT-TCG-GGA-CTT-CTA-CTC-GC | TCA-CA-CAT-ACC-CAG-TCA-GGC |
| ***Cyp2b10*** | NM_009999 | TTC-TGC-CCT-TCT-CAA-CAG-GA | CCT-TAG-GAG-CAA-CAT-GGC-TT |
| ***Cyp3a11*** | NM_007818 | ACG-CCT-CTC-CTT-GCT-GTC-ACC | TTG-CCT-TCT-GCC-TCA-AGT-AC |
| ***Esr1*** | NM_000125;NM_001122740;NM_001122741; NM_001122742 | TGT-TTG-CTC-CTA-ACT-TGC-TC | CCT-TCT-CTT-CCA-GAG-ACT-TC |
| ***Esr2*** | NM_207707; NM_010157 | CTC-TTC-CCA-GCA-GCA-GTC-AGT-C | AGC-ATC-TCC-AGC-AGC-AGG-T |
| ***Fgf15*** | NM_008003 | GAGGAGGACCAAAACGAACG | GAAGGTACAGTCTTCCTCCG |
| ***Fgf21*** | NM_020013 | GGTACCTCTACACAGATGAC | AAGTGAGGCGATCCATAGAG |
| ***Ghr*** | NM_001048178,NM_001048147,NM_010285 | CCTCCATTTGGATACCCTAC | CTCCGTTGTCTGGATCTCAC |
| ***Got2*** | NM_010325 | ATG-GTG-AAG-GAT-GCC-TGG | TTC-ATC-CGC-ATC-TTT-GCA-GAC-C |
| ***Gper1*** | NM_029771 | AGC-TGA-TCA-GAT-CTA-GGG-AG | GTC-CTG-GGA-GCC-TGT-TAG-TC |
| ***Insig2*** | NM_133748;NM_178082;NM_0011271531;NM_001271532 | CTGGAGGCATAACGATGGGA | CGTTGGTGAACCTCTCATAC |
| ***Lepr*** | NM_001122899,NM_010704,NM_146146 | CACAACCGATGACTCCTTTC | CATCCAGCACTCTATGTCC |
| ***Mmp7*** | NM_010810; NM_001319986 | ACG-ACA-TTG-CAG-GCA-TTC-AG | GTT-CAT-GCC-AGC-TGA-GGG-AT |
| ***Muc2*** | NM_023566 | GCT-ATG-ACG-TCT-GTG-TGA-AG | CAA-ACA-CAG-TCC-TTG-CAG-TC |
| ***Ngn3*** | NM_009719 | GCT-ATC-CAC-TGC-TGC-TTG-AC | TGG-AAT-TGG-AAC-TGA-GCA-CTT |
| ***Npc1l1*** | NM_207242 | GCT-AGC-AGC-CAA-CAT-CAC-AG | CAG-TAG-GAG-GTA-GCA-GAC-CA |
| ***Nr1c1*** | NM_011144; NM_001113418.1 | AAG-GGC-TTC-TTT-CGG-CGA-AC | GTT-CAT-GTT-GAA-GTT-CTT-CAG |
| ***Nr1c3*** | NM_001127330,NM_011146 | TCT-CTC-CGT-AAT-GGA-AGA-CC | GCA-TTA-TGA-GAC-ATC-CCC-AC |
| ***Nr1h4*** | NM_001163700.NM_001163504,NM_009108 | GCA-ACC-TGT-TGG-AAG-AAA-G | GTC-TGT-CTG-GAG-AGA-GGA-TG |
| ***Nr1i1*** | NM_009504 | GAATGTGCCTCGGATCTGTGG | ATGCGGCAATCTCCATTGAAG |
| ***Nr1i2*** | NM_010936.3 | AGG-AGG-AGT-ATG-TGC-TGA-TG | CTT-CAG-GAA-CAG-GAA-CCT-GTG |
| ***Nr1i3*** | NM_001243063;NM_001243062;NM_009803 | GTC-CCA-TCT-GTC-CGT-TTG-C | AGG-GCT-TCT-GAC-AGT-ATC |
| ***Nr3c1*** | NM_008173 | ACACCTGGATGACCAAATGACC | GCAGGGTAGAGTCATTCTCTGC |
| ***Nr3c2*** | NM_001083906 | CCAAGTGTCCCAACAGTTCT | CATCTTCCATCACTTCCTGT |
| ***Ocln*** | NM_008756 | CCACCTATCACTTCAGATCAAC | TCAGCAGCAGCCATGTACTC |
| ***Slc10a1*** | NM_001177561,NM_011387 | CTTAAGGACAAGGTGCCCTAC | GTTGCCCACATTGATGACAG |
| ***Slc10a2*** | NM_011388 | ACCTCAGTGTTAGCATGACC | ATCCAGGCACTTTGGTACAG |
| ***Slc2a2*** | NM_031197 | TGG-GTA-CTC-TTC-ACC-AAC-TG | AGG-ATG-TGC-CAA-TGA-TCC-TG |
| ***Slc2a5*** | NM_019741 | ATCACTGTCGGCATCCTTGT | TCTTCTGGATCAGCAGGTAG |
| ***Slc5a1*** | NM_019810 | GCTCCTTGACCTCCATCTTC | CAGGCAATGCTGATGCCAAT |
| ***Tjp1*** | NM_009386 | ACG-CAT-CAC-AGC-CTG-GTT-G | TGG-CTC-CTT-CCT-GTA-CAC-C |
| ***Vnn1*** | NM_ 011704 | CTGAAGTGTTGCTGAGTGAG | GATGCCCAGTCCTTCCCATA |

**Supplemental Figure S1**: Effect of ovariectomy alone (Ovx) or with 17β-estradiol (E2) supplementation (Ovx+E2) or with exposure to pollutants (Ovx + Poll) on the expression of genes encoding xenobiotic receptors in the ileum (A) and the colon (B). Values are means ± SEM with n=6-8. Sham: sham -operated mice.

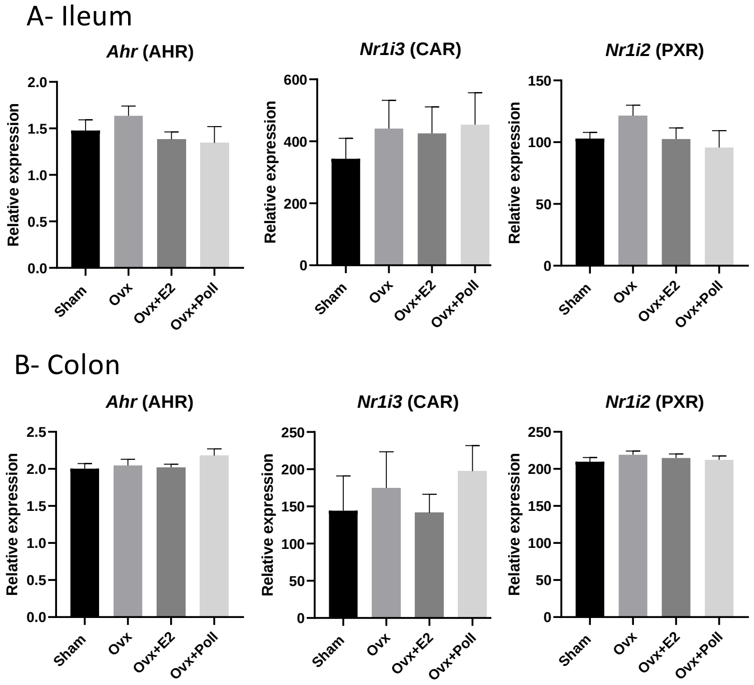


**Supplemental Figure S2**: Effect of ovariectomy alone (Ovx) or with 17β-estradiol (E2) supplementation (Ovx+E2) on the expression of genes encoding xenobiotic receptors and target genes in the liver; and of AHR in the subcutaneous (B) and visceral (C) adipose tissue. Values are means ± SEM with n=6-8. ***P<0.001; ** P<0.01; *P<0.05. Sham: sham -operated mice.

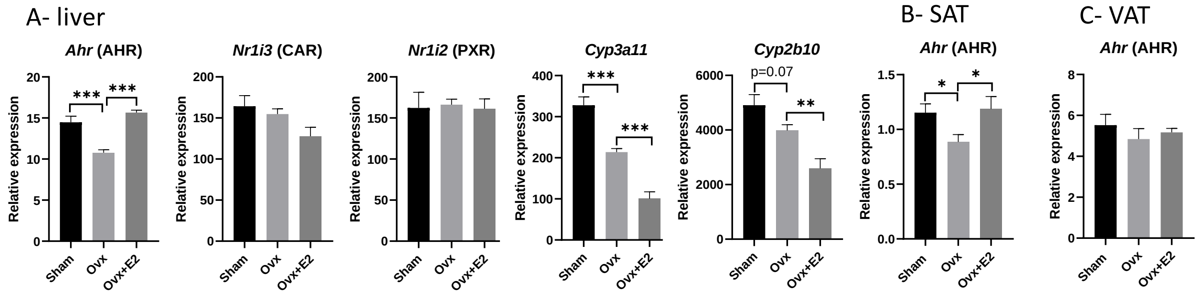

**Supplemental Figure S3**: Effect of ovariectomy alone (Ovx) or with 17β-estradiol (E2) supplementation (Ovx+E2) or with exposure to pollutants (Ovx + Poll) on the expression of the genes measured and found not impacted by the treatments in the ileum. Values are means ± SEM with n=6-8. Sham: sham -operated mice.

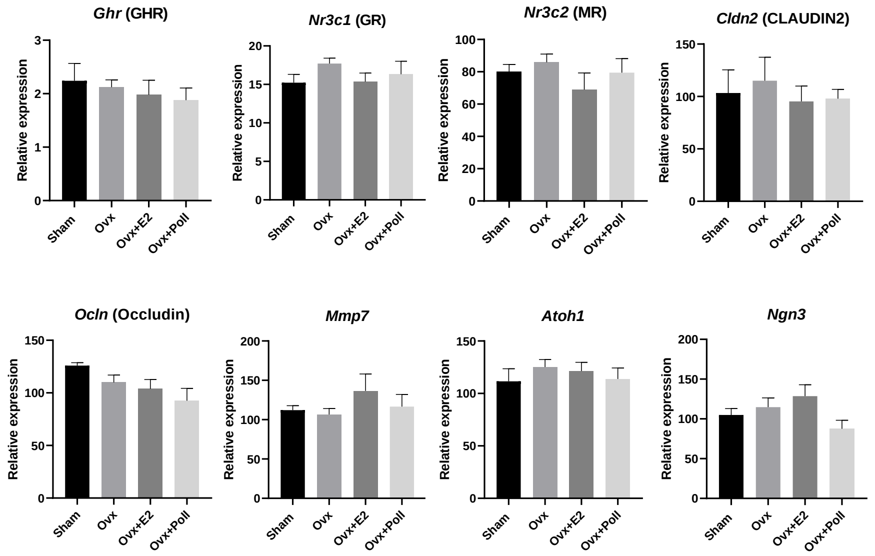


**Supplemental Figure S4**: Effect of ovariectomy alone (Ovx) or with 17β-estradiol (E2) supplementation (Ovx+E2) or with exposure to pollutants (Ovx + Poll) on the expression of the genes measured and found not impacted by the treatments in the colon with the exception of ZO-1 and ATOH-1 (*P<0.05 compared to Ovx mice). Values are means ± SEM with n=6-8. Sham: sham -operated mice.

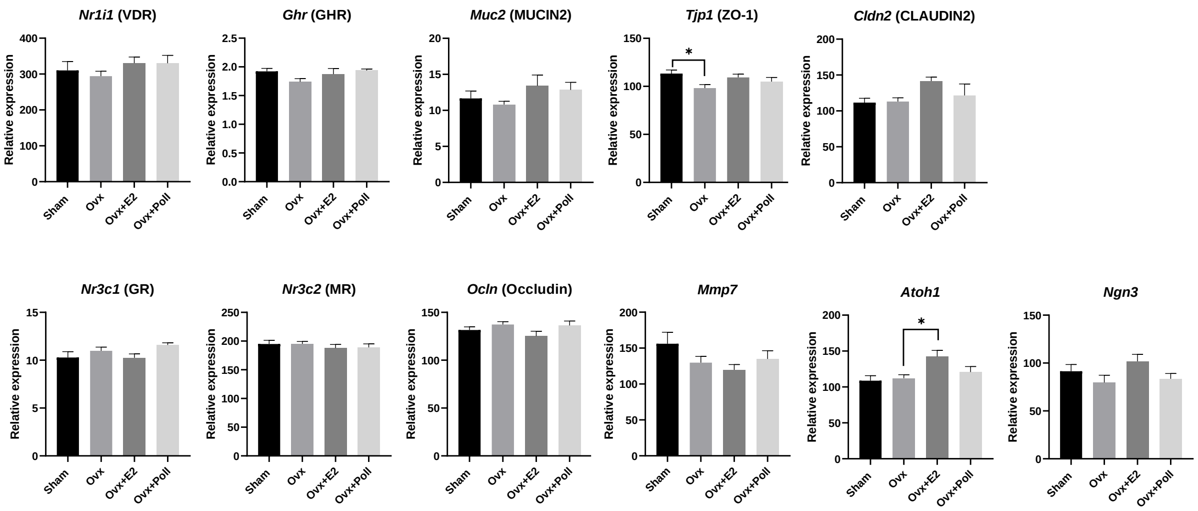

Supplement: Supplementary file 1 [file ijerph-18-08685-s001.zip › ijerph-1267284-supplementary.pdf]
